# The WISDOM Personalized Breast Cancer Screening Trial: Simulation Study to Assess Potential Bias and Analytic Approaches

**DOI:** 10.1093/jncics/pky067

**Published:** 2019-01-08

**Authors:** Martin Eklund, Kristine Broglio, Christina Yau, Jason T Connor, Allison Stover Fiscalini, Laura J Esserman

**Affiliations:** 1 Department of Medical Epidemiology and Biostatistics, Karolinska Intitutet, Stockholm, Sweden; 2 Berry Consultants LLC, Austin, TX; 3 Department of Surgery, University of California San Francisco, San Francisco, CA; 4 Buck Institute for Research on Aging, Novato, CA; 5 University of Central Florida College of Medicine, Orlando, FL; 6 Confluence Stat, Orlando, FL

## Abstract

**Background:**

WISDOM (Women Informed to Screen Depending on Measures of Risk) is a randomized trial to assess whether personalized breast cancer screening—where women are screened biannually, annually, biennially, or not at all depending on risk and age—can prevent as many advanced (stage IIB or higher) cancers as annual screening in women ages 40–74 years across 5 years of trial time. The short study time in combination with design choices of not requiring study entry and exit mammograms for all participants may introduce different sources of bias in favor of either the personalized or the annual arm.

**Methods:**

We designed a simulation model and performed 5000 virtual WISDOM trials to assess potential biases. Each virtual trial simulated 65 000 randomly assigned participants who were each assigned a risk stratum and a time to stage of at least IIB cancer sampled from an exponential distribution with the hazard rate based on the risk stratum. Results from the virtual trials were used to evaluate two candidate analysis strategies with respect to susceptibility for introducing bias: 1) difference between arms in total number of events over total trial time, and 2) difference in number of events within complete screening cycles.

**Results:**

Based on the simulations, about 86 stage IIB or higher cancers will be detected within the trial and the total exposure time will be about 74 000 years in each arm. Potential ascertainment bias is introduced at study entry and exit. Analysis strategy 1 works better for the nonscreened stratum, whereas method 2 is considerably more unbiased for the strata of women screened biennially or every 6 months.

**Conclusion:**

Combining the two candidate analysis approaches gives a reasonably unbiased analysis based on the simulations and is the method we will use for the primary analysis in WISDOM. Publishing the WISDOM analysis approach provides transparency and can aid the design and analysis of other individualized screening trials.

Annual mammograms for women over the age of 40 years have been a key US strategy to reduce breast cancer mortality for 30 years. However, consensus on key aspects of mammography screening, such as the age to begin screening, frequency of screening, and the age to stop screening, have not been reached, and screening guidelines from professional organizations differ (1–5).

Breast cancer risk models incorporating family history of breast disease, endocrine exposures, breast density, and genetic variants are now available (6–9). Armed with this better understanding of breast cancer risk, personalized screening—which tailors screening recommendations, including the starting age, stopping age, frequency, and modality of screening to an individualized risk estimate—has been proposed as an alternative to the current “one-size-fits-all” guideline-based approach (10–12).

The WISDOM (Women Informed to Screen Depending on Measures of Risk) trial is a multicenter, randomized trial comparing personalized screening to annual screening in women ages 40–74 years, initially opening in the Athena Breast Health Network in California and the Midwest (13,14). There are two co-primary study objectives. The first is to test whether personalized screening is safe, as measured by noninferiority in the rate of advanced (stage IIB or higher) cancers. The second is to test whether personalized screening is less morbid, as measured by a reduction in the number of breast biopsies.

Because this screening study has the short-term endpoint of advanced cancers rather than the long-term survival endpoint of mortality, particular care must be taken to avoid biased comparisons between the randomly assigned arms that arise from assessing the women according to different schedules. In this article, we describe the known sources of bias in the comparison of the two randomly assigned arms in the WISDOM trial. We use a simulation study to evaluate the extent of the bias and analytic approaches to reducing the bias. This ultimately informs the structure of the WISDOM primary analyses. Our aim is to provide transparency in our analysis methods of the trial,and to share the tradeoffs made between trial design choices and the potential bias. Moreover, as precision medicine advances, more personalized screening trials will be designed and conducted, and our experiences from designing WISDOM should be useful to others.

## Methods

### WISDOM Design

**Figure 1.**
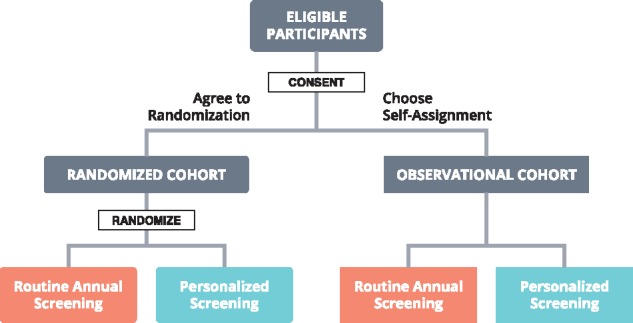
Trial design overview. Women ages 40–74 years with no prior diagnosis of breast cancer or history of bilateral mastectomy are eligible to join the trial. For women who are either randomly assigned to or self-select personalized screening, individualized risk assessment is used to inform screening frequency and modality based on demographical and clinical risk factors using the Breast Cancer Surveillance Consortium risk model (15,16), a polygenic risk score representing the cumulative effects of genetic variants (96 single nucleotide polymorphisms selected based on genome-wide significance in at least one racial or ethnic group: Caucasians, East Asians, Hispanic/Latinos, African Americans) (7,8), and moderate- and high-penetrance germline mutations (BRCA1, BRCA2, TP53, STK11, PTEN, CDH1, ATM, PALB2, and CHEK2) (9).

WISDOM follows a preference-tolerant design (Figure 1) that encourages women to be randomly assigned but also allows self-assignment for those with strong personal preference for either annual or risk-based screening [see eg, Weinstein et al. (17) for a similar design]. For women in the personalized screening arm, individualized risk assessment is used to inform screening frequency and modality [see Shieh et al. (13) for details]. Women’s predicted risk is translated into one of four different screening recommendations (Table 1) (13). Annual mammogram will thus be compared to a mixture of recommendations with different screening intervals. WISDOM is expected to enroll 100 000 women (65 000 into the randomly assigned cohort). The trial time until the first analysis of the primary endpoints is 5.5 years.

**Table 1.** WISDOM risk stratification and screening recommendations (13)

Risk	Highest risk	Elevated risk	Average risk	Lowest risk
Criteria/ threshold	BRCA1/2, TP53, PTEN, STK11, CDH1, ATM, PALB2, or CHEK2 mutation carrier	Women ages 40–49 y with extremely dense breasts	Women ages 50–74 y	Women ages 40–49 y with a <1.3% 5-year risk of developing breast cancer
	or	or	or	
	Women with a ≥6% 5-year risk (risk of an average BRCA carrier)	Women at a ≥1% 5-year risk of developing Estrogen Receptor breast cancer based on susceptibility Single Nucleotide Polymorphisms	Women aged 40–49 y with a ≥1.3% 5-year risk (risk of an average 50-year-old woman)	
	or	or		
	Women with a history of mantle radiation	Women in top 2.5th percentile of risk by 1-year age category		
Screening recommendation	Annual mammogram+ Magnetic Resonance Imaging (MRI)	Annual mammogram*	Biennial mammogram†	No screening until age 50 y

* If individual does not meet criteria for annual mammogram + MRI.

† If individual does not meet criteria for annual mammogram or annual mammogram + MRI. The risk predictions are based on the Breast Cancer Surveillance Consortium risk prediction model together with a polygenic risk score.

### Potential Sources for Bias

#### Study Entry

**Figure 2.**
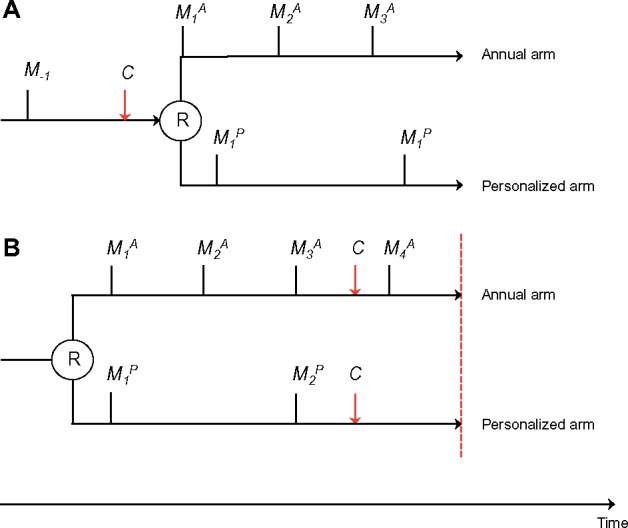
An example woman of average risk is randomly assigned to either the annual arm or to the personalized arm. Black vertical lines indicate mammograms (*M*). R denotes randomization (study entry). Red dashed line indicates end-of-study. Red arrows indicate development of stage IIB or higher breast cancer.

WISDOM does not require a study entry mammogram. Upon enrolling in the trial and receiving a screening recommendation, each woman’s next future mammogram will be scheduled to be consistent with her assigned screening frequency based on the date of her most recent mammogram (Figure 2). Women who enter the trial and are randomly assigned to the personalized arm and receive a “no screening” recommendation will not receive an on-study mammogram unless, during the trial, they meet a threshold for risk that would trigger screening. As a result of not requiring a study entry mammogram, some women will enter the trial with a prevalent cancer. It should be noted, however, that even if a study entry mammogram was used, some women would still enter the study with a prevalent cancer because the sensitivity of mammograms is not 100%. The prevalent cancers can introduce bias in the comparison between the study arms because there is a delay from when participants enter the study to the time when they are screened in accordance with their assigned screening schedule. This delay could be overall longer in the personalized arm compared with the annual arm, resulting in fewer events or longer times to event. However, the actual extent and direction of this bias depends on how prevalent cancers are handled in the analysis (Box 1).

The rationale for not requiring a study entry mammogram is that a key purpose of the trial is to avoid mammograms in the lower risk groups. The goal of the study is to evaluate the initiation of mammographic screening depending on risk. Adding a study mammogram at entry changes the trial intervention to be an initial mammogram followed by a recommended frequency based on risk. In essence, we sacrifice some internal study validity for greater generalizability, which we consider a reasonable design trade-off given the aims and pragmatic nature of the trial.

#### Study Exit

WISDOM will not require a study exit mammogram in general. The exception is women in the personalized arm who received a “no screening” recommendation who will receive an exit mammogram if they have been enrolled in the trial for at least 2 years. Because of the short trial duration, the observed rate of cancers in this group will be assessed to determine if the rate of cancers truly is low or if we observed a low rate because cancers simply were not detected due to lack of screening. The exit mammogram is thus required for this group to avoid verification bias, which otherwise potentially could bias against the annual arm.

The lack of exit mammogram introduces a period of time between last on-study screen and study exit; on average, more time will pass from the last within-study mammogram to the end of the study for women who are screened less frequently compared with women who are screened more frequently (Box 1). Depending on how the trial is analyzed, this may introduce bias in comparisons between the two arms.

### Candidate Analyses

The primary analysis will be based on the randomly assigned cohort only. To have sufficient power, the primary analysis of the safety endpoint will be based on a difference in the risk of stage IIB or higher cancers between the two arms (18). The noninferiority margin is 0.05% absolute difference between the randomly assigned arms. Therefore, the null hypothesis is that personalized screening increases the risk for stage IIB or higher cancers by at least 0.05%.

Below we describe the candidate approaches to analyzing the results with respect to the primary efficacy endpoint.

#### Total Trial Time

The simplest analysis approach is to compare the number of stage IIB cancers diagnosed in each arm throughout the entire trial duration. In this approach, exposure time is counted from the date of study entry to the date of stage IIB cancer detection or date of study exit. We quantify the risk for stage IIB cancers in each arm as a hazard rate or the number of events within this exposure time. The advantage of this approach is that it is transparent, and all stage IIB events, all exposure times, and all randomly assigned women can be included in the analysis. However, this approach does not make any attempt to address the biases introduced at study entry and exit.

#### Time Within Complete Screening Cycles

For this approach, we try to control the potential bias at study exit by counting events and exposure time only within complete screening cycles. Exposure time is counted from the date of study entry to the date of stage IIB cancer detection or last completed screening cycle. Among women in the personalized arm assigned to no screening, there is no screening cycle. Therefore, to consider complete screening cycles among these women, we include only those women who receive a study exit mammogram and consider their screening cycle as the date from study entry to the date of exit mammogram.

For the personalized arm, there will be more exposure time within complete screening cycles for women with shorter screening intervals than for women with longer intervals, because, as an example, three whole annual cycles (= 3 years of exposure time) fit in 3.5 years of follow-up whereas only one whole biennial screening cycle (= 2 years of exposure time) fits into the same time period. Because women undergoing 6 months or annual screening are a higher risk population, the difference in exposure time needs to be corrected for in the analysis by weighting the results within each stratum against the inverse of the total exposure time in the stratum.

Time within complete screening cycles analysis option can be extended to also address the biased introduced at study entry by excluding prevalent cancers from the analysis in both arms. This option is analytically equivalent to giving all women a study entry mammogram.

### Simulation Methods

We used simulation to quantify bias and to understand the performance of the candidates for the primary analysis. We simulate 65 000 virtual women in the trial. The 65 000 virtual women are accrued within 3.5 years. Accrual rates are 5000, 25 000, 20 000, and 15 000 total women in the first 6 months of accrual and then each year of accrual respectively. Women are equally randomly assigned to annual or personalized screening. Independent of randomly assigned arm, women are assigned a risk stratum. We assigned risk stratum randomly according to a multinomial distribution where there is a 28.9%, 40.4%, 28.2%, and 2.5% probability of being in the lowest, average, elevated, and highest risk strata, respectively. Annual hazards for the risk strata were 0.0198%, 0.0414%, 0.0774%, and 0.2808% for women from the lowest to highest risk group, respectively. The proportion of women in each risk stratum and the corresponding annual hazard rates were estimated by applying the Breast Cancer Surveillance Consortium risk model and simulated polygenic risk score (assuming independence between the polygenic risk and other risk factors) to data collected within the Athena Breast Health Network.

We then employ a simple natural history model of breast cancer. First, we randomly assign women to have a prevalent stage IIB cancer at randomization according to Bernoulli trials with an .05% probability. Women who do not have a prevalent stage IIB cancer at randomization are assigned a time to mammogram-detectable stage IIB breast cancer. Time to mammogram-detectable stage IIB cancer is simulated for each woman according to an exponential distribution with the hazard rate depending on risk strata. Sojourn times are then simulated from an exponential distribution such that the median sojourn time is 18 months. The time to clinically detectable stage IIB cancer for each woman is then the sum of her time to mammographically detected cancer and her sojourn time.

Each virtual woman is then assigned a first planned screening time according to her screening recommendation based on the randomization assignment and risk strata. However, we also allow noncompliance to the planned screening time in the form of a delay beyond the planned time. For each planned screening, a noncompliance time is simulated from a truncated normal distribution with a mean (SD) of 0 (4.5) months. This distribution is consistent with 55% of women having their mammogram within 3 months of the scheduled date and 87% having their mammogram within 6 months of the scheduled date (19,20). The actual screening time is then planned mammogram time plus any noncompliance time. Subsequent screenings are then planned to occur according to her recommended schedule relative to the actual screening time, not the planned screening time, and continue to incorporate noncompliance as described. For example, a woman enrolled in the first year of the trial and assigned to annual screening will have planned mammograms 1, 2, and 3 years after her enrollment. Due to noncompliance, her first mammogram may occur at 1 year and 2 months. The next mammogram would then be planned to occur at 2 years and 2 months, but due to noncompliance may occur at 2 years and 6 months, and so on.

The total trial time is 4.5 years. Most women in the trial will not develop a stage IIB cancer during the trial. Women that do not develop a stage IIB cancer during the trial are considered to not have cancer, with their exposure time being from their entry into the trial to the time of their last screen or the time of trial end depending on the analysis.

For women who do develop a stage IIB cancer during the trial, we compare her natural history to her screening schedule. If a screening occurs between the mammogram-detectable and clinically detectable times, the cancer is considered detected by mammogram at the time of screening. Otherwise, if a cancer becomes clinically detectable in the interval between screenings, it is considered clinically detected at that time. The simulations thus assumed 100% sensitivity of a mammogram. To explore the impact of this assumption, we also performed simulations with lower mammogram sensitivity: 93%, 96%, 76%, and 86% for the lowest to highest risk groups, respectively (different sensitivities due to the fact that risk strata are differently associated with BIRADS breast density categories). Table 2 summarizes the parameters used for the simulations.

**Table 2.** Summary of parameters and distributions used in simulations

Parameter	Distribution
Accrual rate	Year 1: 5000
	Year 2: 25 000
	Year 3: 20 000
	Year 4: 15 000
Randomization	1: 1
Proportion in each risk strata	Low risk: 28.9
	Average risk: 40.4%
	Elevated risk: 28.2%
	High risk: 2.5%
Annual hazard for mammogram detected cancer	Low risk: 0.0198
	Average risk: 0.0414
	Elevated risk: 0.0774
	High risk: 0.2808
Median time from mammogram to clinically detectable cancer	18 months
Noncompliance time	Normal^+^ (0, 4 months)
Mammographic sensitivity	100%
	(Low risk: 93%
	Average risk: 86%
	Elevated risk: 76%
	High risk: 86%
	used in sensitivity analyses)
Percent with prevalent stage IIB	0.05%
Total trial time	4.5 y

We simulated the WISDOM trial in the manner described above 5000 times. We track the total number of events and total exposure time in each strata and overall. Exposure time was calculated for the total duration of the trial and among complete screening cycles only, in accordance with the two candidate analyses. All 5000 trial simulations were performed under the alternative hypothesis that personalized screening is equivalent to annual.

## Results

The main results from the simulations are summarized in Tables 3 and 4. Based on our simulations, about 86 stage IIB and higher cancers will be detected within the trial and the total exposure time will be about 74 000 years in each arm. We can expect approximately 33 prevalent and 53 incident stage IIB cancers, and 34 clinically and 52 screen-detected cancers. The total number of mammograms in each arm will be around 42 570 and 26 780 in the annual and personalized arm, respectively (Table 4).

**Table 3.** Number of stage IIB or higher cancers by mode of detection*

	Prevalent cancers stage IIB or higher, no.		Screen detected, no.		Clinically detected, no.		Total, no.	
Risk group	Personalized	Annual	Personalized	Annual	Personalized	Annual	Personalized	Annual
Every 6 months	2.3	2.3	5.1	3.9	1.5	2.1	6.5	6.0
Annual	7.1	7.1	12.0	12.0	6.7	6.6	18.7	18.6
Biennial	5.4	5.4	5.0	9.2	7.1	5.1	12.1	14.3
No screening at this time	1.8	1.8	2.1	3.1	2.9	1.7	5.0	4.9
All risk groups	16.6	16.6	24.2	28.2	18.1	15.5	42.3	43.7

*The table shows number of expected stage IIB or higher cancers in the Women Informed to Screen Depending on Measures of Risk (WISDOM) trial, by mode of detection, risk strata, and analysis approach. The results are based on the simulation model and are averaged across 5000 simulations.

**Table 4.** Expected results from the WISDOM trial

Risk group	Cancer ≥ stage IIB, no.		Exposure time, y		Hazard rate		Mean risk difference		*P*(R < 0)*	Mammograms, no.	
	Personalized	Annual	Personalized	Annual	Personalized	Annual	(95% confidence interval)			Personalized	Annual
Total trial time											
High	6.5	6.0	1851	1850	0.352	0.324	−0.028	(−0.393 to 0.343)	.56	2035	1058
Elevated	18.7	18.6	20 950	20 948	0.089	0.089	0.000	(−0.059 to 0.056)	.50	11 997	12 000
Average	12.1	14.3	30 028	30 031	0.040	0.048	0.007	(−0.026 to 0.040)	.33	6978	17 206
Low	5.0	4.9	21 494	21 492	0.023	0.023	−0.001	(−0.028 to 0.028)	.52	5776	12 311
Overall†	42.3	43.7	74 324	74 320	0.057	0.059	0.002	(−0.023 to 0.026)	.44	26 786	42 576
Time within complete screening cycles											
High	6.4	5.6	1520	1320	0.419	0.424	0.005	(−0.456 to 0.481)	.49	2035	1058
Elevated	17.4	17.4	14 935	14 933	0.117	0.116	0.000	(−0.080 to 0.076)	.50	11 997	12 000
Average	9.4	13.3	15 649	21 405	0.060	0.062	0.003	(−0.049 to 0.051)	.46	6978	17 206
Low	4.5	4.5	16 798	15 320	0.027	0.029	0.003	(−0.034 to 0.041)	.43	5776	12 311
Overall†	37.6	40.8	48 901	52 978	0.077	0.077	0.000	(−0.032 to 0.035)	.45	26 786	42 576
Time within complete screening cycles with entry mammogram											
High	4.3	3.7	1518	1322	0.281	0.282	0.000	(−0.394 to 0.400)	.49	2843	1868
Elevated	11.6	11.7	14 930	14 931	0.078	0.078	0.000	(−0.062 to 0.061)	.50	21 154	21 154
Average	6.5	9.0	15 649	21 401	0.042	0.042	0.000	(−0.041 to 0.042)	.50	20 102	30 327
Low	4.5	4.2	16 794	15 318	0.027	0.027	0.000	(−0.030 to 0.033)	.47	5776	21 707
Overall†	26.9	28.6	48 891	52 973	0.055	0.054	0.000	(−0.027 to 0.027)	.51	49 875	75 055

* The table shows the expected total number of stage IIB or higher cancers, total exposure time, and hazard rate in the Women Informed to Screen Depending on Measures of Risk (WISDOM) trial, by study arm, risk strata, and analysis approach. Computed risk differences between the annual and personalized arm with 95% confidence intervals are also shown. *P*(R < 0) shows the estimated probability that the risk difference is less than 0. Deviations from .5 indicate bias (in favor of the annual arm *P*[R < 0] > .5 and vice versa if *P*[R < 0] < .5). The results are based on the simulation model and are averaged across 5000 simulations.

† Overall results are weighted against the inverse of the total exposure time in each stratum.

### Bias Due to Study Entry

To determine the bias created from the lack of an entry mammogram, we can compare results from simulations when no women receive an entry mammogram and when all women receive an entry mammogram (Table 4, B and C). A study entry mammogram to remove prevalent cancers from the study population clearly results in an almost entirely unbiased trial (the risk difference between personalized and annual screening is very close to 0 in all risk strata; Table 4, C). However, lack of entry mammograms only creates a relatively small bias for the risk strata that are recommended screening in the personalized arm (Table 4, B).

### Bias Due to Study Exit

To determine the bias created from the lack of exit mammogram, we can compare results of the hazard as calculated with the total trial time vs the complete screening cycles (Table 4, A and B).

Using total trial time creates bias in the high- and average-risk strata. In the high-risk strata, the bias favors the annual arm and in the average risk strata the bias favors the personalized arm. The estimated probability that the risk difference for high-risk women in the personalized arm compared with the annual arm is smaller than 0 (*P*[R < 0]) is .56 for high-risk women and .33 for average-risk women. There is no bias introduced for the women with elevated risk, because these women are recommended the same screening schema in both arms. The bias also appears to be low in the stratum where there is no screening in the personalized arm (*P*[R < 0] = .52).

Limiting the analysis to complete screening cycles reduces the bias between arms in the strata with high and average risk. *P*(R < 0) is .49 for high-risk women and .46 for average-risk women. Using our definition of a screening cycle for nonscreened women increases bias using complete screening cycles compared with total trial time (*P*[R < 0] = .43 and *P*[R < 0] = .52, respectively). The price for the reduced bias for the high- and average-risk strata is increased variance. The exposure time counted is decreased by about 25 000 person-years (34%) and 21 000 person-years (29%) in the personalized and the annual arm, respectively, compared with using total trial time. The total number of events is, however, reduced by only 10% and 7%, respectively. Because not all events occurring within the trial are counted towards the primary analysis, the confidence intervals are slightly wider compared with using the total trial time.

Because analyzing complete screening cycles introduces less bias than total trial time for high- and normal-risk women whereas total trial time works better for nonscreened women, a reasonable approach is to combine the analysis methods in a hybrid approach where complete screening cycles are used for the risk strata that are recommended screening in the personalized arm and total trial time is used for the nonscreened women. Doing that results in *P*(R < 0) = .47 across all risk groups (compared with .51 if prevalent cancers are removed using a study entry mammogram).

Lowering the mammogram sensitivity did not materially affect the results of the simulations (data not shown).

## Discussion

Analyses of WISDOM that are as unbiased as possible are crucial for drawing correct conclusions from the trial. We used simulations to evaluate two approaches to analyzing the WISDOM trial: 1) total number of events over the total trial duration, and 2) number of events within complete screening cycles. It is evident from the simulation results that method 1 works better for the nonscreened strata, whereas method 2 is considerably more unbiased for the groups of women screened biennially or every 6 months. Combining the two approaches gives a reasonably unbiased analysis approach based on the simulation results and is the method we will use in WISDOM. The main purpose of the simulations was to understand how WISDOM design choices may create bias and how different analysis choices may correct for some of that bias rather than in a detailed fashion model the natural history of breast cancer, for example, the CISNET consortium (21).

Nonetheless, as observed in the simulation results, undetected stage IIB cancers in women enrolling in the trial (prevalent cancers) does create potential for bias. Informed by the simulation results, we will therefore perform two sensitivity analyses in WISDOM to address this issue. First, exclude cancers detected at first on-study screens. This mimics having a study entry mammogram and provides higher internal validity to the cost of excluding events from the analysis. Second, repeated random exclusion of potential excess cancers detected at first screen compared with subsequent screens (where the screening interval is controlled within the trial).

We have here focused on two potential analysis paths, but many more are possible. For instance, the trial could be analyzed within a model-based time-to-event framework with censoring at the date of a breast cancer diagnosis, loss to follow-up, death, and either at the end of the study or at the time point of the last complete screening cycle. A difficulty with a time-to-event analysis of WISDOM is that the relative hazards are not proportional over trial time (22). Time-varying effects could potentially be used to address this issue; however, it would require fitting a complex and less transparent model to limited data.

This report discussed the analysis of WISDOM’s primary safety endpoint. The same issues with potential bias introduced at study entry and exit arise in the analysis of the primary efficacy endpoint and the same approaches are applicable for the analysis.

Biases introduced at study entry and exit are more pronounced in a comparably short screening trial like WISDOM. However, the benefit of short studies using a relevant proxy endpoint are that they minimize the chance of being obsolete by the end of the trial and enable more rapid dissemination of the results. Thus, it is important for the analytic tools to adjust and support rapid knowledge turns and to keep pace with the continuous advances in the field of risk prediction and breast cancer screening.

Our goal was to provide the background needed to support new designs for modern screening studies and to make the analysis of WISDOM transparent, in particular because it is outside the standard screening trial framework of comparing screening to no screening or of comparing different screening modalities (22–25). As precision medicine advances, the attention to personalized screening is likely to increase, because it is a potentially effective means to balance the benefit and harms of screening. We hope that this report raises the awareness of some possible pitfalls in the analysis and interpretation of personalized screening trials, which are likely to become more common.

## Funding

This work was supported by the Patient-Centered Outcomes Research Institute and the Robert Wood Johnson Foundation. The WISDOM Study is funded by the Patient-Centered Outcomes Research Institute (PCS-1402-10749). The statistical planning began during the pilot and planning phase, which was funded by the Robert Wood Johnson Foundation.

## Notes

Affiliations of authors: Department of Medical Epidemiology and Biostatistics, Karolinska Intitutet, Stockholm, Sweden (ME); Berry Consultants LLC, Austin, TX (KB); Department of Surgery, University of California San Francisco, San Francisco, CA (CY, ASF, LJE); Buck Institute for Research on Aging, Novato, CA (CY); University of Central Florida College of Medicine, Orlando, FL (JC); Confluence Stat, Orlando, FL (JC). 

WISDOM is registered at ClinicalTrials.gov (NCT02620852).

BOX 1. Examples of bias introduced at study entry and exit

To illustrate bias introduced at study entry (**A**) and exit (**B**), we use an example woman of average risk (Figure 2). She is randomly assigned to either the annual arm or to the personalized arm, where she will be screened biennially because of her average risk. The same types of biases occur also for women with very high or very low risk, who are differentially screened in the annual and the personalized arm.

**A.** *Example of bias introduced by prevalent cancers at study entry.*

A woman of average breast cancer risk has a negative mammogram at *M_-1_*. Then 18 months later she decides to enroll in WISDOM. Between her negative mammogram at *M_-1_* and her enrollment in WISDOM, she has developed undetected stage IIB breast cancer. If she is randomly assigned to the annual arm, she will get a mammogram right after enrollment ($M_{1}^{A}$) because her last mammogram was more than 12 months ago. Her prevalent cancer will then likely be diagnosed at $M_{1}^{A}$. However, if she is randomly assigned to the personalized arm, she will be scheduled for biennial mammograms receive her first on-study mammogram 6 months after enrollment ($M_{1}^{P}$), where her prevalent cancer will be diagnosed (if it has not already been symptomatically detected as an interval cancer prior to the mammogram).

*Analysis options:*

1. The prevalent cancer is counted in the primary analysis irrespective of whether the woman is randomly assigned to the annual or the personalized arm. We then risk biasing against the annual arm because the cancer is detected more quickly in the annual arm due to the shorter screening cycle.
2. The prevalent cancer is excluded from analysis in both arms. This option is analytically equivalent to giving all women a study-entry mammogram. This analysis would reflect an intervention that is an initial mammogram followed by a recommended frequency based on risk rather than the actual trial intervention. It is also difficult to determine whether the cancer was stage IIB prevalent at the time of randomization when detected after the time of randomization.

**B.** *Example of bias introduced at study exit.*

A woman of average breast cancer risk has enrolled in WISDOM. She develops stage IIB cancer before the end of the study. If she is in the annual arm, her cancer is detected at $M_{4}^{A}$. However, if she is in the personalized arm, the cancer has a higher chance of remaining undiagnosed until the end of the study, because she will not have another on-study screen after she develops cancer and before the end of the trial.

*Analysis options:*

1. Count exposure time until end of study. Bias is introduced, because more exposure time is added without disease verification if the woman is screened biennially compared with annually.
2. Only counting exposure time within complete screening cycles avoids introducing bias to the cost of not counting all cancers and all exposure time within the trial.

## Supplementary Material

Supplementary Data
