# Supplementary material for: The WISDOM Personalized Breast Cancer Screening Trial: Simulation Study to Assess Potential Bias and Analytic Approaches
Source: JNCI Cancer Spectr. 2019 Jan 8;2(4):pky067. doi: 10.1093/jncics/pky067 (PMC6649825; doi:10.1093/jncics/pky067)
Supplement: Supplementary Data [file pky067_supp.docx]

**Supplement**

**
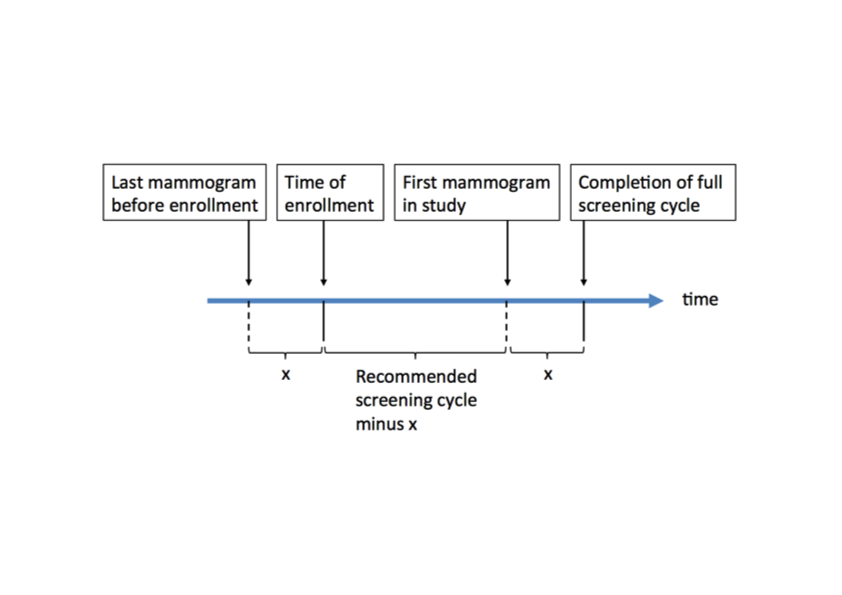
**

**Figure S1**

Upon enrolling in the trial and receiving a screening recommendation, each woman’s next future mammogram will be scheduled. This will be dated from the date of her most recent mammogram in the two years prior to enrollment into the trial. For example, if a woman received a mammogram 1 year prior to study entry and is given a biennial screening recommendation, her first on-study mammogram would be 1 year into the trial (2 minus X, where X in this example is equal to 1). Thus, her first on study mammogram is two years from her last mammogram and is consistent with the biannual recommendation. Alternatively if the same woman received an annual screening recommendation, her first on study mammogram would be scheduled at study entry to be consistent with the annual recommendation. A completed full screening cycle equates to all exposure time during one round of screening, regardless of at what time point in the cycle the clock for counting exposure time starts (if the clock is started X years into the cycle, then exactly X years of exposure time after the last screening occasion is needed in order to complete a full screening cycle).
